# Thalassemia and assisted reproduction: non-transfusion-dependent thalassemia shows no significant effect on live birth rates after embryo transfer

**DOI:** 10.3389/fcell.2025.1573572

**Published:** 2025-03-21

**Authors:** Li Fan, Liuyan Wei, Ni Tang, Zhetao Li, Wugao Li, Liuying Nong, Jingjing Li, Wenjie Huang

**Affiliations:** ^1^ Department of Reproductive Medicine, Guangzhou Women and Children’s Medical Center Liuzhou Hospital, Liuzhou, Guangxi, China; ^2^ Department of Reproductive Medicine, Liuzhou maternity and Child Healthcare Hospital, Liuzhou, Guangxi, China; ^3^ Guangxi Clinical Research Center for Obstetrics and Gynecology, Liuzhou, Guangxi, China; ^4^ Liuzhou Key Laboratory of Gynecologic Tumor, Liuzhou, Guangxi, China

**Keywords:** non-transfusion-dependent thalassemia, thalassemia carriers, live birth rate, assisted reproductive technology, clinical pregnancy

## Abstract

**Background:**

Thalassemia is a hereditary blood disorder that can impact fertility due to various factors such as iron overload and endocrine disruption. While the effects of iron overload on fertility outcomes in transfusion-dependent thalassemia (TDT) have been well-documented, there is limited data on how NTDT affects assisted reproductive technology (ART) outcomes. This study aims to assess the fertility and pregnancy outcomes of NTDT patients compared to thalassemia carriers (TC) patients in IVF and frozen embryo transfer (FET) cycles.

**Methods:**

This retrospective cohort study analyzed 6,911 female patients who underwent autologous IVF treatment at a private reproductive center between January 2013 and December 2022. The study included women who were carriers of thalassemia or diagnosed with NTDT. ART outcomes, including oocyte retrieval rate, embryo development (maturation rate, number of fertilized oocytes and blastocyst formation rate), clinical pregnancy rate, live birth rate, and miscarriage rate, were compared between NTDT and TC patients. Propensity score matching (PSM) and multivariable adjustments for potential confounders were applied in the statistical analyses.

**Results:**

NTDT patients had a significantly lower oocyte retrieval rate (0.88 vs. 0.93, p < 0.05) and a longer interval from medication initiation to oocyte retrieval (13.35 days vs. 12.38 days, p < 0.05) compared to TC patients. However, NTDT patients exhibited higher oocyte maturation rates and a greater number of fertilized oocytes. Despite these differences in embryo development metrics, there were no statistically significant differences in clinical pregnancy rates and live birth rates between NTDT and TC patients in both fresh embryo transfer (IVF-ET) and FET cycles (p > 0.05). These findings suggest that while NTDT may affect certain aspects of embryo development, it does not significantly impact overall pregnancy outcomes in ART.

**Conclusion:**

This study provides valuable insights into ART outcomes for NTDT patients, showing that, despite challenges in oocyte retrieval, their fertility and pregnancy outcomes are comparable to those of thalassemia carriers. Clinicians should consider individualized treatment plans and provide comprehensive counseling for NTDT patients, focusing on their specific fertility characteristics, to optimize ART outcomes. Further research is needed to explore the underlying mechanisms affecting embryo development in NTDT patients and to confirm these findings in broader populations.

## Introduction

Recent advances in techniques like embryo culture, vitrification, genetic screening, and cycle splitting have led to significant breakthroughs in assisted reproductive technology (ART), notably improving treatment success rates, especially cumulative live birth rates (CLBR per ITT) (Esteves et al., 2019). As a result, patient consultations have evolved. With the maturation of genetic screening, patients now focus not only on pregnancy success but also on selecting the healthiest embryos to maximize outcomes and minimize treatment risks (Madero et al., 2023).

Thalassemia is a common hereditary disorder affecting around 5% of the global population, with higher carrier rates in regions like China, India, Southeast Asia, and the Mediterranean, presenting a significant public health challenge (Rund, 2016). It is classified into α-thalassemia and β-thalassemia, with α-thalassemia including Hb Bart syndrome and HbH disease, and β-thalassemia encompassing major, intermedia, and minor forms. Carriers typically exhibit mild microcytic anemia, which does not lead to significant health issues and usually requires no treatment (Langer et al., 1993; Tamary et al., 1993). Thalassemia is further categorized into transfusion-dependent thalassemia (TDT) and non-transfusion-dependent thalassemia (NTDT). TDT patients, mainly those with severe β-thalassemia, need regular blood transfusions to manage anemia and prevent complications, often experiencing endocrine disorders due to iron overload, including menstrual irregularities and infertility (Musallam et al., 2021; Castaldi and Cobellis, 2016). NTDT patients, including those with β-thalassemia intermedia and HbH disease, do not require transfusions but still experience ineffective red blood cell production and iron metabolism issues (Musallam et al., 2021). Research on how these conditions affect fertility in NTDT patients is limited, making it crucial to understand their impact for clinical counseling and treatment decisions.

Third-generation IVF technology (preimplantation genetic diagnosis, PGD) is crucial in assisted reproduction for high-risk thalassemia patients, allowing for the selection of embryos free from genetic diseases, thus reducing the risk of affected offspring and improving treatment success (Ren et al., 2024). According to expert consensus (Yan et al., 2023), PGD is recommended when one or both partners are carriers of thalassemia gene mutations and at risk of having affected children. Due to the high costs of PGD and the relatively low genetic risk in NTDT patients, who often have mild symptoms, most of these patients undergo embryo culture and transfer using first- or second-generation IVF technologies. However, the impact of NTDT on embryo development and pregnancy outcomes in assisted reproduction is not well understood.

In this retrospective cohort analysis, we included all patients who underwent autologous IVF at the Reproductive Medicine Center of Liuzhou Hospital, Guangzhou Women and Children’s Medical Center, from January 2013 to December 2022, with thalassemia gene carriers serving as a control group. The aim is to assess whether NTDT affects fertility and provide data to optimize treatment strategies. A better understanding of thalassemia’s impact on assisted reproduction will help healthcare providers set more accurate expectations, improve patient compliance, and enhance treatment outcomes.

## Materials and methods

This is a retrospective cohort data analysis conducted at an infertility center in southeastern China. The study population consisted of patients who underwent autologous *in vitro* fertilization (IVF) treatment at the Reproductive Medicine Center of Liuzhou Hospital, Guangzhou Women and Children’s Medical Center, between January 2013 and December 2022. The treatment cycles included fresh IVF-embryo transfer (IVF-ET) and frozen embryo transfer (FET). Inclusion criteria for the cycles were as follows: female patients who were either carriers of thalassemia or non-transfusion-dependent thalassemia (confirmed by hematological testing). Cycles involving male patients who were thalassemia carriers or non-transfusion-dependent thalassemia, as well as those using oocyte donation, sperm donation, or frozen-thawed oocytes, were excluded from the study. The study was approved by the Ethics Committee of Liuzhou Hospital, Guangzhou Women and Children’s Medical Center (No. 2024-238).

Patients’ ages were obtained from their identity card information, while the duration of infertility was self-reported by the patients. Details about the infertility diagnosis, ovarian stimulation protocol, and fertilization methods were recorded by the attending physicians. Height and weight were measured during the clinic visit, and body mass index (BMI) was calculated as weight (kg) divided by height (m) squared. Detailed information related to embryos, including the grade and number of embryos transferred, was recorded by the reproductive laboratory. Serum hormone levels were obtained from the clinical laboratory and genetic laboratory databases.

### Stimulation protocol

Patients underwent ovarian stimulation, transvaginal oocyte retrieval, and subsequent blastocyst cryopreservation based on the treatment physician’s judgment. During the treatment, either an antagonist protocol or an agonist protocol was used. The treatment plan included subcutaneous injections of gonadotropins (Gn), with follicular development monitored via transvaginal ultrasound and serum estradiol, luteinizing hormone (LH), and progesterone levels to assess ovarian response. For oocyte maturation, human chorionic gonadotropin (hCG) and/or gonadotropin-releasing hormone agonist (GnRH-a) were administered. Specific treatment protocols and medication use followed established standard procedures from prior literature (Fan et al., 2022), with adjustments made based on individual patient characteristics.

### Fertilization and embryo culture

In this study, fertilization was performed using either conventional insemination or intracytoplasmic sperm injection (ICSI) at the discretion of the treating physician. In the conventional fertilization process, semen samples were prepared under a laminar flow hood, with 0.8–1 mL per well, and the insemination time was adjusted according to follicular maturity and other factors, typically occurring 4–6 h after oocyte retrieval. The sperm concentration was calculated at 50,000 sperm per oocyte, ensuring that no more than six oocytes were inseminated per well. Granulosa cells were removed 4–5 h after fertilization, and viable oocytes were transferred into culture medium. For ICSI, morphologically normal sperm were selected, immobilized, and injected into MII-stage oocytes using micromanipulation techniques. After injection, the cytoplasm was aspirated to ensure the sperm entered the oocyte. All fertilized oocytes were transferred into culture medium and briefly cultured in an incubator. Embryo development was assessed 16–18 h after fertilization. Embryo transfer typically took place 24–48 h post-fertilization, following routine IVF procedures.

### Fresh and thawing embryo transfer

In this study, patients underwent either fresh cycle embryo transfer or FET, based on the judgment of the treating physician. In the fresh cycle, patients received ovarian stimulation, and ovulation was triggered with hCG when follicles reached appropriate size. After oocyte retrieval, fertilization was performed, and the embryos were cultured to the blastocyst stage. The appropriate embryos for transfer were selected based on their development, with transfer typically occurring on day 3 after oocyte retrieval. However, in some cases, blastocysts on day 5 or 6 were transferred depending on the circumstances. For FET, patients underwent embryo transfer at least 2 months after the fresh cycle. Endometrial preparation for FET could involve a natural cycle, clomiphene citrate-induced cycle, or hormone replacement therapy. Once the endometrium reached the appropriate thickness, embryos were thawed and evaluated. Only embryos with a survival rate of over 50% were considered for transfer. A maximum of two embryos or blastocysts were transferred per FET cycle.

### Outcome variables

Various cycle outcomes were examined, including the number of oocyte retrievals, the number of oocytes retrieved, oocyte retrieval rate (oocytes retrieved per punctured follicle), the number of mature oocytes, maturation rate (the percentage of mature oocytes per retrieval cycle), the number of fertilized oocytes, fertilization rate (fertilization rate per mature oocyte), the number of blastocysts cultured, blastocyst formation rate, the time from stimulation to oocyte retrieval (in days), and the time from stimulation to pregnancy (in months). The primary outcomes included pregnancy rate, live birth rate, miscarriage rate in NTDT patients compared to TC, as well as pregnancy rate, live birth rate, and miscarriage rate following frozen embryo transfer. Clinical pregnancy was defined as the observation of an intrauterine gestational sac via ultrasound in early pregnancy, or the recording of live birth, ectopic pregnancy, or miscarriage. Live birth was defined as the birth of one or more live infants. Miscarriage was defined as the loss of an intrauterine pregnancy before 20 weeks of gestation.

### Covariates

The covariates in this study included several baseline factors in women that could influence IVF or intracytoplasmic sperm injection (ICSI) cycles, cycle outcomes, and FET-related parameters. Female covariates included age, BMI, anti-Müllerian hormone (AMH) levels, serum estradiol (E2), follicle-stimulating hormone (FSH), luteinizing hormone (LH), the number of embryos transferred, duration of infertility, fertilization method (IVF or ICSI), infertility type (primary or secondary), infertility diagnosis (e.g., tubal factor, male factor, diminished ovarian reserve, endometriosis, etc.), the timing of embryo transfer (day 3, day 5/6), and ovarian stimulation protocol (agonist, antagonist, mild stimulation, natural cycle, etc.). For FET, covariates included female age, BMI, the number of embryos transferred, duration of infertility, fertilization method (IVF or ICSI), infertility type (primary or secondary), timing of embryo transfer (day 3, day 5/6), and endometrial preparation method (natural cycle or programmed cycle). These covariates were assessed to evaluate the impact of various factors on pregnancy outcomes and were analyzed as potential confounders.

### Propensity score matching (PSM)

To reduce confounding and balance baseline characteristics between the two study groups, PSM was used. PSM is a statistical technique that matches individuals from different groups based on their estimated probability (propensity score) of receiving a treatment, given their baseline characteristics. In this study, a 1:2 matching ratio was used to match NTDT with TC patients based on several baseline covariates. The propensity scores were estimated using logistic regression, and patients who could not be matched were excluded from the analysis. This method helped balance the groups, making the comparison of ART outcomes between NTDT and TC patients more robust and minimizing confounding bias.

## Statistical analysis

All data analyses were performed using SPSS 26.0 and the R statistical language in RStudio, with a two-sided significance level set at 0.05. There were no missing data for outcome variables, and the missing rate for other variables was below 5%. For missing data, the median imputation method was applied, which is suitable for skewed data, to minimize the impact of extreme values and ensure the representativeness of the data.

For statistical comparisons, Student’s t-test and Mann-Whitney U test were used to compare continuous variables, while the chi-square test was used for categorical variables. For the analysis of pregnancy outcomes, we first fitted an unadjusted model and then further fitted a multivariable-adjusted model, incorporating all baseline factors into the adjusted model to control for potential confounders. To further explore the relationship between pregnancy outcomes and various covariates, we performed interaction effect analysis to assess the interactions between different covariates. All relevant covariates were included in the interaction effect model to analyze their potential impact on pregnancy outcomes.

## Results

From January 2013 to December 2022, a total of 6,911 patients from the Reproductive Medicine Center met the inclusion criteria. Among them, 2,908 patients (42.1%) underwent fresh embryo transfer cycles, while 4,002 patients (57.9%) underwent FET cycles. In the fresh embryo transfer cycles, 2,346 patients were in the TC group, and 562 patients were in the NTDT group. In the FET cycles, 2,995 patients were in the TC group, and 1,007 patients were in the NTDT group.

### Propensity score matching

Table 1 shows the baseline characteristics before and after propensity score matching (PSM) for 13 baseline factors. The data are reported on a per-cycle basis. In both the fresh embryo transfer and FET cycles, 1:2 propensity score matching was performed between the TC and the NTDT group. In the fresh embryo transfer cycles, 13 variables were matched, including: female age, BMI, AMH, E2, FSH, LH, number of embryos transferred, infertility duration, fertilization method, type of infertility, infertility diagnosis, day of transfer, and ovarian stimulation protocol. In the FET cycles, 8 variables were matched, including: female age, BMI, number of embryos transferred, infertility duration, fertilization method, type of infertility, day of transfer, and endometrial preparation (Supplementary Table S2).

**TABLE 1:** Baseline characteristics of women undergoing fresh cycles for TC and NTDT groups before and after 1:2 propensity score matching.

Baseline characteristic	Overall, n = 2908	Before matching		SMD	After matching		SMD
		TC, n = 2346	NTDT, n = 562		TC, n = 1124	NTDT, n = 562	
Age	34.08 ± 4.98	34.30 ± 5.05	33.15 ± 4.60	0.237	33.60 ± 4.71	33.15 ± 4.60	0.097
BMI (kg/m2)	21.90 ± 3.00	22.02 ± 3.04	21.41 ± 2.77	0.208	21.50 ± 2.86	21.41 ± 2.77	0.032
AMH (ng/mL)	2.48 ± 2.14	2.68 ± 2.22	1.65 ± 1.47	0.544	1.79 ± 1.28	1.65 ± 1.47	0.097
E2 (pg/mL)	47.46 ± 61.66	47.59 ± 67.49	46.89 ± 25.81	0.014	45.64 ± 35.68	46.89 ± 25.81	0.040
FSH (mIU/mL)	6.64 ± 2.78	6.48 ± 2.64	7.31 ± 3.22	0.285	7.03 ± 2.94	7.31 ± 3.22	0.094
LH (mIU/mL)	3.76 ± 2.36	3.74 ± 2.46	3.85 ± 1.91	0.049	3.73 ± 2.33	3.85 ± 1.91	0.057
No. of embryos transferred	1.60 ± 0.51	1.56 ± 0.51	1.73 ± 0.51	0.336	1.70 ± 0.49	1.73 ± 0.51	0.073
Infertility duration (y)	4.94 ± 3.97	4.89 ± 4.01	5.15 ± 3.82	0.066	5.10 ± 4.10	5.15 ± 3.82	0.013
Fertilization method (%)				0.084			0.033
IVF	2247 (77.3)	1797 (76.6)	450 (80.1)		885 (78.7)	450 (80.1)	
ICSI	661 (22.7)	549 (23.4)	112 (19.9)		239 (21.3)	112 (19.9)	
Type of infertility (%)				0.188			0.101
Primary	949 (32.6)	725 (30.9)	224 (39.9)		393 (35.0)	224 (39.9)	
Secondary	1959 (67.4)	1621 (69.1)	338 (60.1)		731 (65.0)	338 (60.1)	
Infertility diagnosis (%)				0.146			0.037
Tubal factor	2055 (70.7)	1645 (70.1)	410 (73.0)		815 (72.5)	410 (73.0)	
Male factor	591 (20.3)	479 (20.4)	112 (19.9)		226 (20.1)	112 (19.9)	
Diminished ovarian reserve	66 (2.3)	59 (2.5)	7 (1.2)		18 (1.6)	7 (1.2)	
Endometriosis	48 (1.7)	35 (1.5)	13 (2.3)		23 (2.0)	13 (2.3)	
Other	148 (5.1)	128 (5.5)	20 (3.6)		42 (3.7)	20 (3.6)	
Day of transfer (%)				0.136			0.011
Day 3	2145 (73.8)	1704 (72.6)	441 (78.5)		877 (78.0)	441 (78.5)	
Day 5/6	763 (26.2)	642 (27.4)	121 (21.5)		247 (22.0)	121 (21.5)	
Ovarian stimulation protocol, No. (%)				0.408			0.072
Agonist	2104 (72.4)	1640 (69.9)	464 (82.6)		928 (82.6)	464 (82.6)	
Antagonist	618 (21.3)	553 (23.6)	65 (11.6)		142 (12.6)	65 (11.6)	
Mild Stimulation	121 (4.2)	112 (4.8)	9 (1.6)		19 (1.7)	9 (1.6)	
Natural cycles	13 (0.4)	10 (0.4)	3 (0.5)		6 (0.5)	3 (0.5)	
Other	52 (1.8)	31 (1.3)	21 (3.7)		29 (2.6)	21 (3.7)	

Data are presented as mean ± SD, for continuous variables and as number (%) for categorical variables. SMD, standardized mean difference; after PSM, no statistically significant differences were observed between the TC, and NTDT, groups for any baseline variables (all p > 0.05). TC, thalassemia carrier; NTDT, Non-Transfusion-Dependent Thalassemia; Day 3 = cleavage-stage embryos; Day 5/6 = blastocyst-stage embryos; FSH, follicle-stimulating hormone; IVF, *in vitro* fertilization; ICSI, intracytoplasmic sperm injection; AMH, anti-Müllerian hormone; E2 = estradiol; LH, luteinizing hormone; BMI, body mass index.

In the fresh cycle, before matching, there were significant differences between the two groups in variables such as female age (SMD = 0.237), BMI (SMD = 0.208), AMH (SMD = 0.544), FSH (SMD = 0.285), and ovarian stimulation protocol (SMD = 0.408). After matching, the SMD values for these variables significantly decreased to less than 0.1, indicating improved balance in baseline characteristics between the two groups (Table 1). In the frozen-thawed cycle, before matching, there were differences between the two groups in variables such as female age (SMD = 0.120), number of embryos transferred (SMD = 0.404), and fertilization method (SMD = 0.201). After matching, the SMD values for these variables also significantly decreased (e.g., number of embryos transferred SMD = 0.078, fertilization method SMD = 0.043), further improving the balance between the two groups (Supplementary Table S2).

### Cycle characteristics and embryo outcomes

Table 2 reports the cycle characteristics and embryo outcome data after applying the propensity score model, comparing TC group with NTDT group. The NTDT group had a significantly longer interval between the start of medication and oocyte retrieval compared to the TC group (13.35 days vs. 12.38 days, p < 0.001), but their oocyte retrieval rate was significantly lower than that of the TC group (0.88 vs. 0.93, p = 0.018). Moreover, the NTDT group had a significantly higher maturation rate than the TC group (1 vs. 0.94, p = 0.015), and they also had a significantly greater number of fertilized oocytes (6 vs. 5, p = 0.005). Although both groups showed similar outcomes for most embryo parameters (such as blastocyst formation rate and number of blastocysts), the time interval from ovarian stimulation to the first positive hCG result was significantly longer in the NTDT group compared to the TC group (0.97 months vs. 0.93 months, p < 0.001).

**TABLE 2:** Cycle characteristics and embryonic outcomes in thalassemia carriers and non-transfusion-dependent thalassemia patients after propensity score matching.

Cycle characteristics	TC, n = 1124	NTDT, n = 562	p
Follicle punctured	9 (6–13)	10 (7–14)	0.006
Oocytes retrieved	9 (5–13)	9 (6–13)	0.299
Oocyte retrieval rate (per follicle punctured)	0.93 (0.71–1.12)	0.88 (0.67–1.11)	0.018
Mature oocytes	7 (5–11)	8 (5–11)	0.072
Maturation rate (per oocyte retrieved)	0.94 (0.8–1)	1 (0.83–1)	0.015
Fertilized oocytes	5 (3–8)	6 (3–9)	0.005
Fertilization rate (per mature oocyte)	0.75 (0.58–0.88)	0.75 (0.6–0.89)	0.09
Blastocysts cultured	6 (4–8)	6 (4–9)	0.077
Blastocyst formation	3 (1–5)	3 (2–5)	0.167
Blastocyst formation rate	0.5 (0.33–0.71)	0.5 (0.33–0.7)	0.799
Time from stimulation initiation to oocyte retrieval (days)	12.38 (10.43–13.4)	13.35 (11.4–14.38)	<0.001
Time from stimulation initiation to pregnancy (months)			
Time to first positive hCG result	0.93 (0.9–1)	0.97 (0.9–1)	<0.001
Time to first live birth	8.83 (8.56–9.07)	8.83 (8.58–9.07)	0.731

Data are presented as median (interquartile range, IQR). TC, refers to patients identified as carriers of thalassemia-related gene mutations. NTDT, refers to patients diagnosed with thalassemia but not dependent on regular blood transfusions. Statistical comparisons between groups were performed using the Mann-Whitney U test due to non-normal data distribution. Time intervals are expressed in days (stimulation to oocyte retrieval) or months (stimulation to pregnancy). p-values <0.05 were considered statistically significant.

Additionally, we conducted an interaction analysis between baseline variables and embryo outcomes for the TC and NTDT groups (Supplementary Table S1). The results indicated that lower AMH levels (interaction estimate −0.4566, p = 0.0089) and lower E2 levels (interaction estimate −0.0179, p = 0.0404) were significantly associated with fewer punctured follicles in the NTDT group. Furthermore, lower E2 levels were significantly associated with a longer oocyte retrieval duration in the NTDT group (interaction estimate −0.0095, p = 0.0163). Moreover, the effect of FSH levels on maturation rate showed a significant interaction between the two groups (interaction estimate −0.0068, p = 0.0036). Other baseline variables, such as BMI and LH, did not show significant interactions (p > 0.05).

### Pregnancy outcomes

Table 3 summarizes the pregnancy outcomes for TC group and NTDT group in both fresh embryo transfer cycles and FET cycles. In the fresh embryo transfer cycles, the clinical pregnancy rate and live birth rate were slightly higher in the TC group compared to the NTDT group (52.3% vs. 47.9%, 44.8% vs. 41.1%), but the differences were not significant (aRR = 0.90, 95% CI: 0.78–1.05; aRR = 0.90, 95% CI: 0.77–1.06). The miscarriage rate was similar between the two groups (7.6% vs. 6.8%, aRR = 0.93, 95% CI: 0.63–1.36). In the FET cycles, the pregnancy outcomes in the TC group and NTDT group were also similar. The clinical pregnancy rate and live birth rate in the TC group were 49.9% and 39.6%, respectively, while in the NTDT group, they were 47.9% and 39.0%. After adjustment, the differences remained statistically insignificant (aRR = 0.94, 95% CI: 0.84–1.05; aRR = 0.97, 95% CI: 0.86–1.09). The miscarriage rate was slightly lower in the TC group compared to the NTDT group (9.3% vs. 7.7%, aRR = 0.83, 95% CI: 0.64–1.09). Interaction analysis results (Supplementary Table S3) also indicated that there was no statistically significant interaction between thalassemia subgroups (TC group vs. NTDT group) and baseline variables on clinical pregnancy outcomes (p > 0.05). This further supports the consistency of the impact of thalassemia carrier status on pregnancy outcomes across different baseline characteristics.

**TABLE 3:** Pregnancy outcomes in fresh and FET cycles for TC and NTDT patients after propensity score matching.

Outcomes	Events, no/total (%)		Relative risk (95% Cl)	
	TC (1124)	NTDT (562)	RR	aRR^a^
Clinical pregnancy	588 (52.3)	269 (47.9)	0.92 (0.82–1.01)	0.90 (0.78–1.05)
Live birth	503 (44.8)	231 (41.1)	0.92 (0.81–1.03)	0.90 (0.77–1.06)
Miscarriage	85 (7.6)	38 (6.8)	0.89 (0.61–1.28)	0.93 (0.63–1.36)
FET	TC (2014)	NTDT (1007)	RR	aRR^b^
Clinical pregnancy	1004 (49.9)	482 (47.9)	0.96 (0.89–1.04)	0.94 (0.84–1.05)^b^
Live birth	797 (39.6)	393 (39.0)	0.99 (0.90–1.08)	0.97 (0.86–1.09)
Miscarriage	188 (9.3)	78 (7.7)	0.83 (0.64–1.06)	0.83 (0.64–1.09)

Outcomes are presented as the number of events/total cases (%). Unadjusted relative risk (RR) was calculated using a generalized linear model with a binomial distribution and log link function. Adjusted relative risk (aRR) were estimated using a Poisson regression model with a log link function, adjusted for potential confounders. Relative risks and 95% confidence intervals were calculated by exponentiating the model coefficients and their respective confidence intervals.

^a^ Adjusted for age, BMI, AMH, E2, FSH, LH, No. of embryos transferred, infertility duration, fertilization method, type of infertility, infertility diagnosis, day of transfer, ovarian stimulation protocol.

^b^ Adjusted for age, BMI, No. of embryos transferred, infertility duration, fertilization method, type of infertility, day of transfer, endometrial preparation.

## Discussion

Thalassemia patients often face unique counseling issues in assisted reproductive treatment, particularly regarding embryo health, pregnancy success rates, and genetic risks. These concerns tend to exacerbate patients’ anxiety, especially after multiple failed transfers or miscarriages, which can lead to increased psychological burden. Therefore, providing scientifically grounded and objective fertility counseling has become a pressing challenge. The aim of this study is to provide valuable information regarding NTDT patients in the assisted reproductive process, addressing the gap in existing literature. We analyzed data from 6,911 patients and found that, although the oocyte retrieval rate was lower in the NTDT group, several embryonic development indicators were superior to those in the TC group. Further analysis revealed that the NTDT group had lower pregnancy outcomes (clinical pregnancy rate and live birth rate) compared to the TC group, although the differences did not reach statistical significance. Clearly, the advantage of the NTDT group in embryonic development did not fully translate into higher clinical pregnancy and live birth rates, which may be related to specific physiological mechanisms, differences during embryo transfer, or uterine environment factors.

The reasons for female infertility in thalassemia patients are multifactorial. One of the most common causes is the interference of iron overload with the endocrine system. Iron accumulation due to long-term blood transfusion therapy can lead to hypogonadotropic hypogonadism (HH) (Castaldi and Cobellis, 2016), resulting in menstrual absence and infertility. One mechanistic hypothesis suggests that oxidative stress (OS) caused by iron overload may affect the hypothalamic-pituitary-ovarian axis, thus inhibiting ovarian function (Roussou et al., 2013). Studies have shown that free radicals produced by iron overload can lead to oxidative damage to cell membranes and DNA, which not only affects the endocrine system but may also cause abnormal follicular development (Devine et al., 2012). In our study, the oocyte retrieval rate in the NTDT group was significantly lower than that in the TC group (0.88 vs. 0.93, P = 0.018), which may be attributed to follicular development abnormalities caused by iron overload. Additionally, iron accumulation can cause dysfunction in endocrine glands, particularly the hypothalamus and pituitary, leading to insufficient secretion of gonadotropins (LH, FSH) (Roussou et al., 2013), which in turn affects ovarian hormone synthesis and ovarian reserve function. Our interaction analysis results also support this mechanism, as low AMH levels (interaction estimate −0.4566, p = 0.0089) and low E2 levels (interaction estimate −0.0179, p = 0.0404) were closely associated with a decrease in follicle count in the NTDT group, suggesting that endocrine dysfunction may be an important factor contributing to the lower oocyte retrieval rate in the NTDT group.

Although iron overload-induced oxidative stress exacerbates the negative effects on oocyte maturation, embryo cleavage, and blastocyst formation (Gao et al., 2023; Deng et al., 2020), as observed in previous studies, the findings in the context of thalassemia major patients with iron overload are not entirely consistent. Studies have shown that, despite ovarian dysfunction in women with thalassemia major, the fertilization rate and cleavage rate during in IVF are actually higher than in the control group, with no significant difference in oocyte quality, such as the formation rate of high-quality embryos (Mensi et al., 2019). Interestingly, embryos carrying thalassemia genotypes even demonstrated better development than normal embryos (Piyamongkol et al., 2012). Our study also confirms these findings: the oocyte maturation rate and fertilized oocyte count in the NTDT group were significantly higher than those in the TC group, with no significant difference in blastocyst formation rate between the two groups. This suggests that, despite the potential oxidative stress caused by iron overload in the NTDT group, its impact on oocyte maturation and fertilization is more complex and may be modulated by other factors.

The situation is more complicated when it comes to implantation and subsequent pregnancy, as these processes involve the timely interaction between the embryo and the endometrium, both of which are regulated by multiple complex signaling pathways and interactions between cells and between cells and the extracellular matrix (Hannan et al., 2011; Tepekoy et al., 2015; Kurian and Modi, 2019). In our study, both in fresh and frozen-thawed embryo transfer cycles, the clinical pregnancy rate and live birth rate in the NTDT group were lower than those in the TC group, although the differences did not reach statistical significance. Existing literature offers an interesting perspective on this trend: excess iron accumulation may alter the uterine microenvironment by inducing oxidative stress and local inflammatory responses, thereby reducing endometrial receptivity (Li et al., 2021; Chen et al., 2021). Under normal conditions, the endometrium needs to maintain proper energy metabolism and cell viability to support successful embryo implantation. However, iron overload may cause mitochondrial dysfunction, reducing ATP production and impairing the metabolism and survival of endometrial cells (Gao et al., 2019). Additionally, iron accumulation is closely associated with apoptosis and iron-dependent cell death (ferroptosis), which not only reduces the number of suitable endometrial cells but may also inhibit cell proliferation and differentiation, further impairing endometrial receptivity (Ng et al., 2020). However, current evidence is primarily based on mouse models and is largely focused on iron overload research. Therefore, future studies need to further validate and expand these findings in a broader clinical population to better understand the specific role of iron overload in pregnancy outcomes in different contexts.

The clinical implications of the study findings are as follows: 1) The NTDT group exhibited a higher oocyte maturation rate, but the oocyte retrieval rate was lower, and the retrieval time was longer. Clinically, it is important to tailor treatment plans according to the specific condition of NTDT patients, optimizing oocyte retrieval and embryo quality. 2) Although there may be differences in oocyte retrieval rates and embryo quality in NTDT patients, pregnancy outcomes in both fresh embryo transfer cycles and frozen embryo transfer cycles were not significantly affected. Therefore, patients should not overly worry when choosing an embryo transfer plan, but rather focus on factors such as medical history, ovarian reserve, and hormone levels. Nevertheless, this study has limitations. First, as a retrospective analysis, we are unable to establish causality and can only reveal associations between different subgroups. Future prospective studies are needed to more clearly define the causal relationships between these factors and pregnancy outcomes. Second, despite the use of propensity score matching (PSM) to control for 13 baseline factors, there may still be unaccounted confounding factors, such as genetic background or environmental influences, which could limit the generalizability of the results.

## Conclusion

This study provides valuable insights for the treatment of thalassemia patients in assisted reproduction. Although there were no significant differences in pregnancy outcomes between the fresh and frozen embryo transfer cycles in NTDT patients, differences in oocyte retrieval rates and maturation rates suggest the need for individualized treatment plans. For thalassemia patients, assisted reproduction specialists should focus on ovarian response characteristics, provide scientifically based treatment plans, and offer psychological counseling to improve treatment success rates and pregnancy outcomes.

## Data Availability

The raw data supporting the conclusions of this article will be made available by the authors, without undue reservation.
