# Supplementary material for: Thalassemia and assisted reproduction: non-transfusion-dependent thalassemia shows no significant effect on live birth rates after embryo transfer
Source: Front Cell Dev Biol. 2025 Mar 21;13:1573572. doi: 10.3389/fcell.2025.1573572 (PMC11968656; doi:10.3389/fcell.2025.1573572)
Supplement: Supplementary file 1 [file Table1.docx]

**Supplemental Table 1**: Interaction effects between NTDT and baseline covariates on embryonic outcomes.

| **Interaction** | Follicle punctured | | Oocyte retrieval rate | | Maturation rate | | Fertilized oocytes | | Time to oocyte retrieved | |
| --- | --- | --- | --- | --- | --- | --- | --- | --- | --- | --- |
|  | **Estimate** | **p value** | **Estimate** | **p value** | **Estimate** | **p value** | **Estimate** | **p value** | **Estimate** | **p value** |
| Age | -0.0812 | 0.1215 | 3.94E-03 | 0.3796 | -2.54E-03 | 0.1013 | -6.82E-02 | 0.0683 | 9.50E-03 | 0.6899 |
| BMI | 0.0675 | 0.4372 | -0.0036 | 0.631 | 0.0003 | 0.9204 | -0.0217 | 0.7266 | 0.0391 | 0.3219 |
| AMH | -0.4566 | 0.0089 | 9.85E-03 | 0.5093 | 9.09E-03 | 0.0782 | -9.36E-03 | 0.9402 | 2.12E-02 | 0.7888 |
| E2 | -1.79E-02 | 0.0404 | -5.37E-04 | 0.4725 | 1.51E-04 | 0.5592 | -6.18E-03 | 0.3218 | -9.53E-03 | 0.0163 |
| FSH | 0.1270 | 0.1079 | -1.48E-03 | 0.8269 | -6.79E-03 | 0.0036 | 7.60E-02 | 0.1778 | -4.53E-02 | 0.2063 |
| LH | -0.1104 | 0.3605 | 0.0029 | 0.7816 | 0.0041 | 0.2554 | 0.0867 | 0.3139 | -0.0358 | 0.5132 |
| Fertilization method | -0.4222 | 0.4845 | -8.42E-02 | 0.1028 | -1.11E-03 | 0.9506 | -7.57E-01 | 0.0789 | -3.43E-01 | 0.2110 |
| Infertility duration | -0.1089 | 0.0794 | 2.32E-03 | 0.6618 | -2.80E-04 | 0.8788 | -4.86E-02 | 0.2726 | -3.91E-02 | 0.1650 |
| Type of infertility | 0.3363 | 0.5002 | 6.69E-02 | 0.1164 | -1.91E-02 | 0.1957 | 2.41E-01 | 0.4976 | 4.00E-01 | 0.0769 |
| Infertility diagnosis |  |  |  |  |  |  |  |  |  |  |
| Male factor | -0.0086 | 0.9888 | 8.86E-03 | 0.8656 | -0.0046 | 0.7980 | -0.0289 | 0.9473 | -0.2013 | 0.4684 |
| Diminished ovarian reserve | -2.2326 | 0.2951 | 2.35E-01 | 0.1964 | -0.1070 | 0.0891 | -0.8585 | 0.5726 | -0.0133 | 0.9891 |
| Endometriosis | -0.5169 | 0.7540 | -5.00E-02 | 0.7228 | 0.0805 | 0.0983 | 0.7039 | 0.5499 | 1.1495 | 0.1244 |
| Other | -0.3238 | 0.8046 | -2.24E-02 | 0.8414 | -0.0059 | 0.8777 | -0.1045 | 0.9109 | -0.8206 | 0.1669 |
| Ovarian stimulation protocol |  |  |  |  |  |  |  |  |  |  |
| Antagonist | -0.7318 | 0.3322 | -9.36E-02 | 0.1467 | -0.0353 | 0.1125 | -0.4405 | 0.4135 | -0.2754 | 0.4214 |
| Mild Stimulation | -1.6454 | 0.3964 | 2.10E-01 | 0.2045 | -0.0816 | 0.1536 | -0.4059 | 0.7695 | -0.4015 | 0.6484 |
| Natural cycles | -2.0993 | 0.5284 | 1.38E-01 | 0.6288 | -0.0202 | 0.8369 | -0.6639 | 0.7800 | 0.8585 | 0.5700 |
| Other | 1.1724 | 0.3918 | -3.20E-02 | 0.7847 | -0.1170 | 0.0038 | 0.5439 | 0.5779 | -0.7615 | 0.2205 |

FSH = follicle-stimulating hormone; AMH = anti-Müllerian hormone; E2 = estradiol; LH = luteinizing hormone; BMI = body mass index.

**Supplemental Table 2**. Baseline characteristics of women undergoing FET cycles for TC and NTDT groups before and after 1:2 propensity score matching.

| Baseline Characteristic | Overall, n = 4002 | Before matching | | SMD | After matching | | SMD |
| --- | --- | --- | --- | --- | --- | --- | --- |
|  |  | NT, n = 2995 | NTDT, n = 1007 |  | NT, n = 2014 | NTDT, n = 1007 |  |
| Age | 34.15 ± 4.98 | 34.30 ± 5.10 | 33.72 ± 4.60 | 0.120 | 34.30 ± 4.99 | 33.72 ± 4.60 | 0.122 |
| BMI (kg/m2) | 21.72 ± 2.95 | 21.78 ± 2.97 | 21.54 ± 2.90 | 0.079 | 21.72 ± 2.97 | 21.54 ± 2.90 | 0.060 |
| No. of embryos transferred | 1.40 ± 0.52 | 1.35 ± 0.49 | 1.56 ± 0.56 | 0.404 | 1.52 ± 0.52 | 1.56 ± 0.56 | 0.078 |
| Infertility duration (y) | 4.99 ± 3.98 | 5.01 ± 4.02 | 4.93 ± 3.86 | 0.019 | 5.04 ± 4.01 | 4.93 ± 3.86 | 0.026 |
| Fertilization method (%) |  |  |  | 0.201 |  |  | 0.043 |
| IVF | 2901 (72.5) | 2240 (74.8) | 661 (65.6) |  | 1281 (63.6) | 661 (65.6) |  |
| ICSI | 1101 (27.5) | 755 (25.2) | 346 (34.4) |  | 733 (36.4) | 346 (34.4) |  |
| Type of infertility (%) |  |  |  | 0.096 |  |  | 0.088 |
| Primary | 1392 (34.8) | 1007 (33.6) | 285 (38.2) |  | 685 (34.0) | 285 (38.2) |  |
| Secondary | 2610 (65.2) | 1988 (66.4) | 622 (61.8) |  | 1329 (66.0) | 622 (61.8) |  |
| Day of transfer (%) |  |  |  | 0.143 |  |  | 0.004 |
| Day 3 | 1299 (32.5) | 921 (30.8) | 378 (37.5) |  | 752 (37.3) | 378 (37.5) |  |
| Day 5/6 | 2703 (67.5) | 2074 (69.2) | 629 (62.5) |  | 1262 (62.7) | 629 (62.5) |  |
| Endometrial preparation, No. (%) | |  |  | 0.063 |  |  | 0.059 |
| Natural cycle | 1141 (28.5) | 875 (29.2) | 266 (26.4) |  | 585 (29.0) | 266 (26.4) |  |
| Programmed cycle | 2861 (71.5) | 2120 (70.8) | 741 (73.6) |  | 1429 (71.0) | 741 (73.6) |  |

Data are presented as mean ± SD for continuous variables and as number (%) for categorical variables. TC = Thalassemia Carriers; NTDT = Non-Transfusion-Dependent Thalassemia; Day 3 = cleavage-stage embryos; Day 5/6 = blastocyst-stage embryos; BMI = Body Mass Index; IVF = In Vitro Fertilization; ICSI = Intracytoplasmic Sperm Injection; FET = frozen embryo transfer

**Supplemental Table 3**: Interaction effects between NTDT and baseline covariates on clinical pregnancy outcomes.

| **Interaction** | **Estimate** | **Std Error** | **Z-value** | **p value** |
| --- | --- | --- | --- | --- |
| Age | -0.0005 | 0.0165 | -0.028 | 0.9773 |
| BMI | 0.0266 | 0.026 | 1.021 | 0.3071 |
| AMH | -0.02458 | 0.051 | -0.481 | 0.6303 |
| E2 | 8.00E-04 | 2.70E-03 | 0.29 | 0.7721 |
| FSH | -0.04278 | 0.0336 | -1.275 | 0.2025 |
| LH | 0.0079 | 0.0356 | 0.222 | 0.824 |
| No. of embryos transferred | -0.1609 | 0.1463 | -1.1 | 0.2716 |
| Fertilization method | -0.1495 | 0.1889 | -0.791 | 0.4287 |
| Infertility duration | -0.0183 | 0.0214 | -0.854 | 0.3931 |
| Day of transfer | 0.0733 | 0.1658 | 0.442 | 0.6587 |
| Type of infertility | 0.2447 | 0.153 | 1.6 | 0.1097 |
| Infertility diagnosis |  |  |  |  |
| Male factor | -0.184 | 0.19 | -0.971 | 0.331 |
| Diminished ovarian reserve | 0.553 | 0.881 | 0.628 | 0.53 |
| Endometriosis | -0.04 | 0.478 | -0.083 | 0.934 |
| Other | -0.297 | 0.413 | -0.718 | 0.473 |
| Ovarian stimulation protocol |  |  |  |  |
| Antagonist | -4.24E-01 | 2.89E-01 | -1.468 | 0.1421 |
| Mild Stimulation | 7.64E-01 | 1.42E+00 | 0.538 | 0.5907 |
| Natural cycles | 1.47E+01 | 5.14E+02 | 0.029 | 0.9772 |
| Other | -1.90E-01 | 6.52E-01 | -0.291 | 0.7708 |

This table presents the interaction effects between NTDT and covariates on clinical pregnancy outcomes, as analyzed using regression models. Data are presented as Estimate (regression coefficient), Standard Error (Std Error), Z-value, and p value. BMI = body mass index; AMH = anti-müllerian hormone; E2 = estradiol; FSH = follicle-stimulating hormone; LH = luteinizing hormone. No statistically significant interaction effects were observed (p > 0.05 for all variables).
